# Time-of-day at symptom onset was not associated with infarct size and long-term prognosis in patients with ST-segment elevation myocardial infarction

**DOI:** 10.1186/s12967-019-1934-z

**Published:** 2019-05-29

**Authors:** Hendrik B. Sager, Oliver Husser, Sabine Steffens, Karl-Ludwig Laugwitz, Heribert Schunkert, Adnan Kastrati, Gjin Ndrepepa, Thorsten Kessler

**Affiliations:** 1 Klinik für Herz- und Kreislauferkrankungen, Deutsches Herzzentrum München, Technische Universität München, Lazarettstr. 36, 80636 Munich, Germany; 2 Deutsches Zentrum für Herz-Kreislauf-Forschung (DZHK) e.V, Partner Site Munich Heart Alliance, Munich, Germany; 3 Klinik für Innere Medizin I, Kardiologie, St. Johannes-Hospital Dortmund, Dortmund, Germany; 4 Institut für Prophylaxe und Epidemiologie der Kreislaufkrankheiten, Ludwig-Maximilians-Universität München, Munich, Germany; 5 I. Medizinische Klinik und Poliklinik, Klinikum rechts der Isar, Technische Universität München, Munich, Germany

**Keywords:** Circadian rhythm, ST-segment elevation myocardial infarction, Infarct size, Primary percutaneous coronary intervention

## Abstract

**Background:**

ST-segment elevation myocardial infarction (STEMI) displays circadian variability with the highest incidence in the morning hours. Data on whether the time-of-day at symptom onset affects infarct size or patients’ long-term prognosis are conflicting. We sought to investigate the association of time-of-day at symptom onset with infarct size or long-term mortality in patients with STEMI undergoing primary percutaneous coronary intervention (PPCI).

**Methods:**

This study included 1206 STEMI patients undergoing PPCI. All patients underwent single photon emission computed tomography (SPECT) imaging with 99mTc-sestamibi before and 7–14 days after PPCI. The co-primary endpoints were final infarct size on day 10 after STEMI and all-cause mortality at 5-year follow-up. Time-of-day at symptom onset of STEMI was categorized in 6-h intervals.

**Results:**

In patients presenting from 0 to 6 h, 6 to 12 h, 12 to 18 h, and 18 to 24 h, the infarct sizes (median [25th–75th percentiles]) were 10.0 [3.0–24.7], 10.0 [3.0–24.0], 10.0 [3.0–22.0], and 9.0 [3.0–21.0] of the left ventricle, respectively (p = 0.87); the Kaplan–Meier estimates of 5-year all-cause mortality were 13.6%, 8.7%, 13.7% and 9.3%, respectively (log-rank test p = 0.30). After adjustment, time-of-day was not associated with infarct size (p ≥ 0.76 for comparisons with infarct size from reference [6–12 h] time interval) or 5-year all-cause mortality (p ≥ 0.25 for comparisons with mortality from reference [6–12 h] time interval). Time-of-day at symptom onset of STEMI was not associated with differences in the recovery of left ventricular ejection fraction 6 months after STEMI.

**Conclusions:**

In patients with STEMI undergoing PPCI, time-of-day at symptom onset was neither associated with scintigraphic infarct size, left ventricular ejection fraction recovery at 6 months nor with 5-year mortality.

**Electronic supplementary material:**

The online version of this article (10.1186/s12967-019-1934-z) contains supplementary material, which is available to authorized users.

## Background

The biological circadian clock, comprising a central (suprachiasmatic nucleus in the hypothalamus) and peripheral (autonomous networks in peripheral tissues) clock, orchestrates circadian rhythms which are crucial for maintaining cardiovascular physiology. Blood pressure, heart rate, autonomic nervous system activity, release of glucocorticoids, and catecholamines display cyclic variations [1, 2]. Cardiovascular events also show a time-of-day dependence [3–5]. Epidemiological studies have shown that myocardial infarction (MI), angina, ventricular arrhythmias, and sudden cardiac death show daily rhythmicity with a first peak of events in the morning (6–12 h), a second in the evening (18–22 h), and a trough during night (0–6 h) [2]. Furthermore, some studies have suggested an association between time-of-day at symptom onset and the course of disease [6–11]. These studies were motivated by findings that cardiomyocytes exhibit an internal circadian clock and may consequently respond differently to injury at certain times of a day [12, 13].

Preclinical studies revealed that the time-of-day at onset of cardiac ischemia may influence post-MI healing. Experimental studies in mice with permanent coronary ligation showed that circadian oscillations of neutrophil recruitment may affect infarct size, myocardial healing, and cardiac function [14]. Similarly, in ischemia/reperfusion injury studies, the largest infarct size was detected when mice were injured at sleep–wake-transition [15]. Clinical studies investigating the association of time-of-day onset of ischemia with infarct size revealed conflicting results. While some studies reported that infarct size differed according to the time of MI onset [6–10, 16–19], others failed to detect a clear circadian dependence of infarct size after ST-segment elevation myocardial infarction (STEMI) [20]. Obviously, the timing of reperfusion therapy will also affect infarct size which will thus depend on the availability of qualified personnel during night hours.

Data on the clinical relevance of the time-of-day onset of STEMI in terms of prognosis are sparse. In this study, we sought to investigate the association of time-of-day at symptom onset in STEMI patients undergoing primary percutaneous coronary intervention (PPCI) in a tertiary care center with: (1) documented time intervals from symptom onset to hospital admission, (2) scintigraphic infarct size measured with single photon emission computed tomography (SPECT) imaging using 99mTc-sestamibi; and (3) 5-year clinical outcome after PPCI.

## Methods

### Study design

The study details and characteristics of source sample have been described before [21, 22]. Briefly, between January 2002 and December 2007, 1406 patients with STEMI underwent PPCI and paired scintigraphic studies at two tertiary care centers (Deutsches Herzzentrum München and Klinikum rechts der Isar, both Technical University of Munich). The diagnosis of STEMI was based on chest pain lasting ≥ 20 min and persistent ST-segment elevation ≥ 1 mm in at least two extremity or ≥ 2 mm in at least two precordial electrocardiographic leads or new onset of left bundle branch block. The diagnosis of STEMI was confirmed with coronary angiography in all patients. 200 out of 1406 STEMI patients were excluded because the time-of-day at symptom onset was not clearly documented. Consequently, 1206 STEMI patients with scintigraphic data on infarct size were included in this study. By design, the study represents a retrospective analysis. All patients gave written informed consent for the procedures. The study conforms to the Declaration of Helsinki.

### Angiography and PPCI

All procedures were carried out at two tertiary care centers (Deutsches Herzzentrum München and Klinikum rechts der Isar, both Technical University of Munich), with 24 h/7 days PPCI service. The time-of-day at symptom onset as well as the time of hospital admission were routinely recorded in the hospital charts. Coronary angiography, PPCI and periprocedural care were performed as per standard care [21, 22]. The culprit lesion in infarct-related artery was defined in the presence of acute occlusion, intraluminal filling defects (or thrombus), ulcerated plaques with contrast-filled pocket protruding into plaque with or without delayed contrast wash-out, extraluminal contrast, dissection or intraluminal flaps. Coronary artery disease in non-culprit lesions was defined as coronary stenosis of ≥ 50% lumen obstruction. Left ventricular ejection fraction (LV-EF) was measured on left ventricular (LV) angiograms using the area-length method. Unfractionated heparin was used for periprocedural anticoagulation. The antithrombotic regime included clopidogrel, a loading dose of 600 mg, and aspirin 325 to 500 mg. Chronic antithrombotic therapy consisted of clopidogrel, 150 mg until discharge (no more than 3 days) followed by 75 mg/day for ≥ 1 month (mostly) and aspirin 200 mg/day indefinitely.

### Measurement of myocardial area at risk and final infarct size by SPECT

99mTc-sestamibi single-photon computed tomography (SPECT) imaging was performed as described previously [21, 22]. Briefly, SPECT imaging was performed serially in each patient at two time points:

1. 99mTc-sestamibi (27 mCi (1000 MBq)) was intravenously injected before PPCI and imaging was carried out 6 to 8 h after injection. Here, perfusion defect was assessed and represents the myocardial area at risk.
2. 99mTc-sestamibi was again intravenously injected 7 to 14 days after PPCI and imaging was performed 6–8 h thereafter. Here, perfusion defect was determined and represents the final infarct size.

Perfusion defects were defined as < 50% uptake of 99mTc-sestamibi and were expressed as percentage of the left ventricle. Myocardial salvage index was calculated as initial myocardial areal at risk minus final infarct size divided by initial myocardial area at risk. The myocardial salvage index shows the proportion of initial myocardial area at risk salvaged by reperfusion. All measurements were carried out by investigators unaware of clinical or angiographic data.

Creatine kinase myocardial band (CKMB) was measured daily and peak level was defined as the highest in-hospital value. CKMB was used as an enzymatic estimate of infarct size.

### Study outcomes and follow-up

Infarct size in the second scintigraphy and 5-year all-cause mortality were the co-primary endpoints of the study. Cardiovascular mortality, salvage index, nonfatal myocardial infarction, target vessel revascularization, and major adverse cardiovascular events (MACE)—composite of death, nonfatal myocardial infarction or target vessel revascularization—were also analyzed. As a standard practice in our institution at the time of patient recruitment, all patients were scheduled to undergo coronary angiography 6 months after the procedure or whenever they showed symptoms or signs of myocardial ischemia and the angiograms were used to calculate 6-month LV-EF. Nonfatal myocardial infarction was diagnosed based on development of new abnormal Q waves in ≥ 2 contiguous precordial or ≥ 2 adjacent extremity leads, or an elevation of CKMB > 2 times (> 3 times for 48 h after a PCI procedure) the upper limit of normal in the presence of ischemia symptoms. Cardiac deaths were defined according to the Academic Research Consortium criteria [23]. Target vessel revascularization was defined as any repeat PCI or bypass surgery of the target vessel over the follow-up. Follow-up information was obtained by staff members unaware of the clinical data including time-of-day at symptom onset via phone calls 30 days after PCI, 1 year after PCI, and yearly thereafter. Data on mortality were obtained from hospital records, death certificates, or phone contact with patients’ relatives or referring physicians.

### Statistical analysis

Continuous data are presented as mean ± standard deviation or median with 25th–75th percentiles depending on normality of distribution and compared with one-way ANOVA or Kruskal–Wallis test, as appropriate. Discrete variables were presented as proportions (percentages) and compared with Chi square test. The association between time-of-day at symptom onset and infarct size (co-primary endpoint) was tested using the multiple linear regression model (with infarct size as continues variable) or multivariable logistic regression model (with infarct size dichotomized at median value). All variables of Table 1 with a p-value ≤ 0.05 were included in the model. Long-term clinical outcomes were assessed using the Kaplan–Meier method. The association between time of symptom-onset (categorized at 0–6 h, 6–12 h, 12–18 h, and 18–24 h time intervals) and all-cause (or cardiac) mortality (co-primary endpoint) was tested using the Cox proportional hazards models and hazard ratios (HR) with 95% confidence interval (CI) were calculated for 0–6 h, 12–18 h or 18–24 h time intervals vs. 6–12 h time interval which served as reference. All parameters of Additional file 1: Table S1 (for all-cause mortality) and Additional file 1: Table S2 (for cardiac mortality) with a p-value < 0.05 (univariable correlates of all-cause or cardiac mortality) plus infarct size were entered into the model(s). Assuming a HR of 2.0 and a type I error rate of 5%, we calculated a power of 91% using the sample size of n = 1206 patients. IBM SPSS Statistics version 24 and GraphPad Prism 7 (version 7.0a) were used for statistical analysis and visualization of data. Circadian variation in area at risk, final infarct size, and myocardial salvage was assessed using the ‘cosinor’ package in R (Version 3.4.3, R Foundation for Statistical Computing, Vienna, Austria).

**Table 1:** Baseline and procedural characteristics according to time-of-day at symptom onset

Characteristic	Time of symptom onset (hours)				p-value
	0–6 n = 273 (22.6%)	6–12 n = 355 (29.4%)	12–18 n = 309 (25.6%)	18–24 n = 269 (22.3%)	
Age, years	61.4 ± 13	62.0 ± 12.8	62.8 ± 13.6	62.9 ± 12.5	0.45
Women, n (%)	64 (23.4)	100 (28.2)	65 (21.0)	57 (21.2)	0.11
Diabetes mellitus, n (%)	60 (22.0)	62 (17.5)	55 (17.8)	52 (19.3)	0.49
BMI, kg/m^2^	26.2 (24.2;28.9)	26.3 (24.2;29)	26.2 (24.1;28.6)	26.1 (24.2;28.6)	0.85
Hypertension, n (%)	202 (74)	244 (68.7)	220 (71.2)	180 (66.9)	0.29
Hypercholesterolemia, n (%)	163 (59.7)	199 (56.1)	148 (47.9)	140 (52.0)	0.03
Current smoker, n (%)	122 (44.7)	143 (40.3)	127 (41.1)	125 (46.5)	0.37
Prior MI, n (%)	36 (13.2)	38 (10.7)	42 (13.6)	35 (13.0)	0.67
Prior CABG, n (%)	10 (3.7)	14 (3.9)	6 (1.9)	9 (3.3)	0.50
Anterolateral location of MI, n (%)	169 (61.9)	195 (54.9)	199 (64.4)	152 (56.5)	0.05
Killip class ≥ 2, n (%)	76 (27.8)	98 (27.6)	72 (23.3)	65 (24.2)	0.46
ST-segment resolution, %	50.1 (20.4;76)	55.5 (28.5;77.3)	48.1 (22.3;74.3)	56.4 (29.4;76.7)	0.36
GFR, mL/min	86.3 (64.2;108.9)	84.5 (64.4;109.2)	83.3 (63.7;105.9)	82.9 (61.5;106.5)	0.57
Time to admission, hours	6.5 (3.3;11)	3.5 (1.8;6.2)	3.8 (1.8;8.9)	5.0 (2.2;14.3)	< 0.0001
Door to balloon, hours	1.3 (0.9;1.8)	1.2 (0.8;1.6)	1.2 (0.8;1.7)	1.3 (1;1.8)	0.02
Multivessel disease, n (%)	175 (64.1)	218 (61.4)	203 (65.7)	182 (67.7)	0.41
Baseline TIMI flow grade					0.63
0, n (%)	130 (47.6)	169 (47.6)	140 (45.3)	128 (47.6)	
1, n (%)	41 (15.0)	37 (10.4)	35 (11.3)	24 (8.9)	
2, n (%)	57 (20.9)	83 (23.4)	76 (24.6)	63 (23.4)	
3, n (%)	45 (16.5)	66 (18.6)	58 (18.8)	54 (20.1)	
No reflow, n (%)	33 (12.1)	50 (14.1)	48 (15.5)	35 (13.0)	0.66
Type of intervention					0.50
Stenting, n (%)	237 (86.8)	307 (86.5)	261 (84.5)	239 (88.8)	
Balloon angioplasty, n (%)	36 (13.2)	48 (13.5)	48 (15.5)	30 (11.2)	
LV-EF, %	49 (41;57)	51 (44;59)	49 (41;55)	49 (43;56)	0.13
Infarct vessel					0.48
LM, n (%)	1 (0.4)	2 (0.6)	1 (0.3)	0 (0)	
LAD, n (%)	117 (42.9)	153 (43.1)	154 (49.8)	115 (42.8)	
LCx, n (%)	55 (20.1)	50 (14.1)	47 (15.2)	50 (18.6)	
RCA, n (%)	94 (34.4)	144 (40.6)	102 (33.0)	99 (36.8)	
CABG, n (%)	6 (2.2)	6 (1.7)	5 (1.6)	5 (1.9)	
Presentation during office hours	143 (52.4)	275 (77.5)	169 (54.7)	79 (29.5)	< 0.0001

Data are presented as mean ± standard deviation or median (25th–75th percentiles) or counts (%)

*BMI* body mass index, *CABG* coronary artery bypass graft, *GFR* glomerular filtration rate, *LV*-*EF* left ventricular ejection fraction, *LAD* left anterior descending artery, *LCX* left circumflex artery, *LM* left mainstem, *MI* myocardial infarction, *RCA* right coronary artery

## Results

### Baseline data

This study included 1206 STEMI patients. The day was divided into four time intervals: 0–6 h, 6–12 h, 12–18 h and 18–24 h as described previously [16, 20]. Overall 273 patients (22.6%) had a symptom onset at 0–6 h time interval, 355 patients (29.4%) at 6–12 h time interval, 309 patients (25.6%) at 12–18 h time interval and 269 patients (22.3%) at 18–24 h time interval. Baseline characteristics of patients according to time-of-day at symptom onset are shown in Table 1. The baseline variables did not differ in between the groups according to the time-of-day at symptom onset with the exception of frequency of hypercholesterolemia, infarct localization, time-to-admission interval, door-to-balloon time interval, and presentation during office hours.

### Time-of-day at symptom onset and final infarct size

**Fig. 1:**
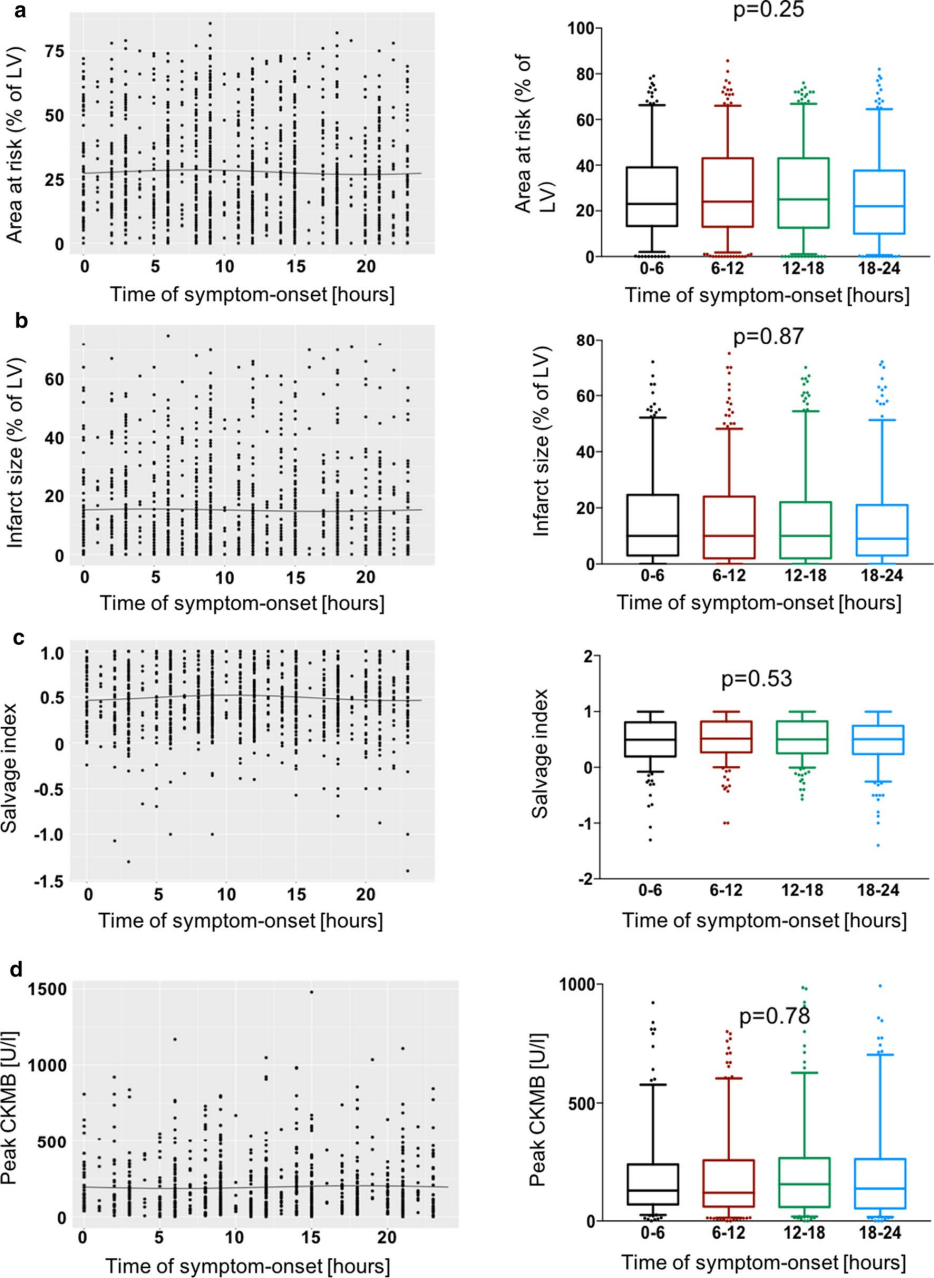
Time-of-day at symptom onset and estimates of myocardial damage. **a** Initial area at risk. **b** Infarct size in the 7 to-14-day scintigraphy. **c** Salvage index—the proportion of initial area at risk salvaged by reperfusion. **d** Creatine kinase myocardial band (CKMB). For each parameter, the left panel shows the distribution of data whereas the right panel shows median values with 25th–75th percentiles. *LV* left ventricle

Scintigraphic data are shown in Table 2 and Fig. 1. None of scintigraphic parameters differed according to the time-of-day at symptom onset. The infarct size (co-primary endpoint) was 10.0 [3.0–24.7], 10.0 [3.0–24.0], 10.0 [3.0–22.0] and 9.0 [3.0–21.0] of the left ventricle in patients with symptom onset at 0–6 h, 6–12 h, 12–18 h, and 18–24 h, respectively. (p = 0.87). In STEMI patients presenting with symptom onset between 0–6 h, 6–12 h, 12–18 h, and 18–24 h, peak CKMB values were: 128 [69–238] U/L, 119 [60–255], 155 [59–265] U/L and 136 [53–260] U/L (p = 0.78, Fig. 1). After adjustment in the multiple linear regression model (see “Methods” for variables entered into the model), time-of-day was not independently associated with infarct size (coefficient = − 0.14; p = 0.67). In a separate analysis, patients were dichotomized into groups based on the median value of initial area at risk, infarct size and salvage index, which were 23.9% of the left ventricle for area at risk, 10.0% for infarct size and 0.5 for salvage index. The association of time-of-day with values ≤ or > median of all these parameters is shown in Table 3. As seen unadjusted or adjusted odds ratios (obtained from multiple logistic regression model) did not show an association between time-of-day (intervals, 0–6 h, 12–18 h and 18–24 h) vs. 6–12 h time interval (reference interval). Of note, an analysis including only individuals presenting within 3 h after symptom onset did also not reveal different results (Additional file 1: Table S3). Moreover, circadian oscillations in blood leukocyte numbers which are normally present in healthy individuals [24, 25] were abrogated after STEMI (Additional file 1: Figure S1).

**Table 2:** Initial area at risk, infarct size and salvage index according to the time-of-day at symptom onset

Parameter	Time-of-day				p-value
	0–6 h	6–12 h	12–18 h	18–24 h	
Initial area at risk (% of the LV)	23.0 [13.4–39.0]	24.0 [13.0–43.0]	25.0 [12.6–43.0]	22.0 [10.0–37.6]	0.25
Infarct size (% of LV)	10.0 [3.0–24.7]	10.0 [3.0–24.0]	10.0 [3.0–22.0]	9.0 [3.0–21.0]	0.87
Salvage index	0.49 [0.19–0.81]	0.52 [0.27–0.82]	0.50 [0.25–0.82]	0.50 [0.24–0.75]	0.53

Data are median with 25th–75th percentiles. Salvage index shows the proportion of initial area at risk salvaged by reperfusion of the initial myocardial area at risk

**Table 3:** Association of time-of-day at symptom onset with area at risk > median, final infarct size > median or salvage index > median

Risk estimate	Time-of-day at symptom onset (hours)				p-value
	0–6 h (n = 273)	6–12 h (n = 355)	12–18 h (n = 309)	18–24 h (n = 269)	
Area at risk > median					
OR (95% CI)	0.916 (0.668–1.256)^1^	Ref	1.075 (0.792–1.459)^2^	0.813 (0.592–1.117)^3^	0.59^1^ 0.64^2^ 0.81^3^
OR_adj*_ (95% CI)	0.871 (0.617–1.231)^1^	Ref	0.990 (0.711–1.380)^2^	0.841 (0.582–1.216)^3^	0.44^1^ 0.95^2^ 0.36^3^
Final infarct size > median					
OR (95% CI)	1.030 (0.751–1.413)^1^	Ref	0.969 (0.714–1.315)^2^	0.916 (0.667–1.257)^3^	0.85^1^ 0.84^2^ 0.59^3^
OR_adj*_ (95% CI)	1.054 (0.757–1.467)^1^	Ref	1.024 (0.722–1.361)^2^	1.024 (0.720–1.456)^3^	0.76^1^ 0.96^2^ 0.89^3^
Salvage index > median					
OR (95% CI)	0.873 (0.633–1.205)^1^	Ref	0.948 (0.694–1.295)^2^	0.994 (0.719–1.375)^3^	0.41^1^ 0.74^2^ 0.97^3^
OR_adj*_ (95% CI)	0.827 (0.591–1.157)^1^	Ref	0.894 (0.647–1.234)^2^	0.913 (0.638–1.305)^3^	0.27^1^ 0.50^2^ 0.62^3^

*Adjusted for hypercholesterolemia, anterolateral location of MI, time to admission, door to balloon time, and presentation during office hours

Data are presented as odds ratios (OR) with 95% confidence interval (CI) with time-of-day at symptom onset 0–6 h serving as a reference (Ref)

^1^0–6 h time interval vs. reference time interval

^2^12–18 h time interval vs. reference time interval

^3^18–24 h time interval vs. reference time interval

### Time-of-day at symptom onset and recovery of left ventricular ejection fraction

**Fig. 2:**
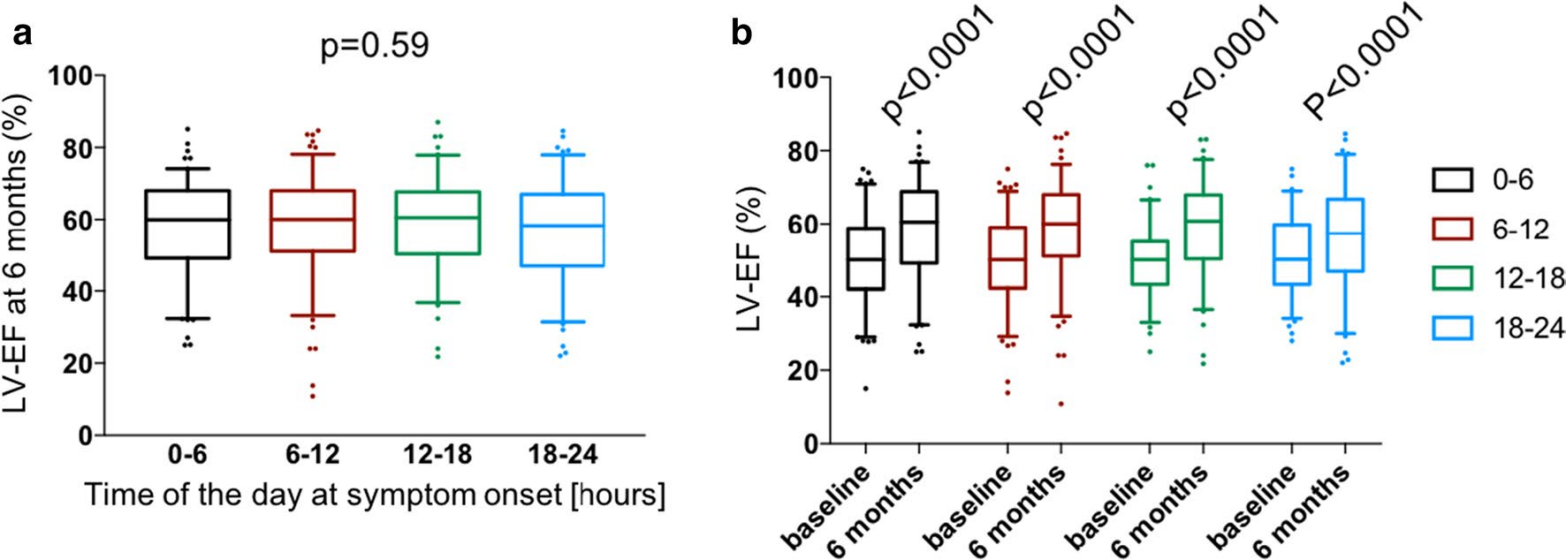
Time-of-day at symptom onset and left ventricular ejection fraction (LV-EF). **a** LV-EF at 6 months. **b** Improvement of LV-EF compared with baseline values. Data are median with 25th–75th percentiles. Numbers: 0–6 h, n = 120; 6–12 h, n = 138; 12–18 h, n = 103; 18–24 h, n = 109

LV-EF data 6-month follow-up were available in 470 patients (39%): 120 patients with a time of symptom onset at 0–6 h, 138 patients with a time of symptom onset at 6–12 h, 103 patients with a time of symptom onset at 12–18 h and 109 patients with a time of symptom onset at 18–24 h. In these groups, the LV-EF values at 6 months follow-up (median with 25th–75th percentiles) were: 60 [49–68]%, 60 [51–68]%, 61 [50–68]%, and 58 [47–67]%, respectively (p = 0.59; Fig. 2a). LV-EF data at baseline and 6-month follow-up were available in 447 patients: 113 patients with a time of symptom onset at 0–6 h, 134 patients with a time of symptom onset at 6–12 h, 101 patients with a time of symptom onset at 12–18 h and 99 patients with a time of symptom onset at 18–24 h. In all 4 groups, LV-EF was improved at 6 months compared with baseline values (Fig. 2b).

### Time-of-day at symptom onset and long-term clinical outcomes

**Fig. 3:**
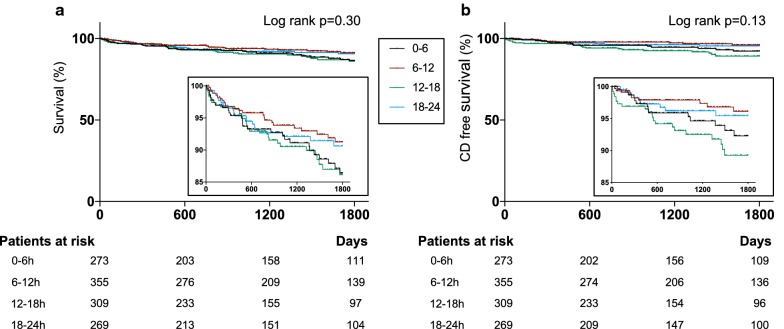
Kaplan–Meier survival curves free from all-cause (**a**) and cardiac mortality (**b**) *CD*, cardiac death

The median follow-up for all-cause death was 1341 days and a complete 5-year follow-up was available in 441 (37%) patients. Overall there were 104 deaths during the follow-up: 28 deaths in patients with a time-of-day at symptom onset at 0–6 h, 24 deaths in patients with a time-of-day at symptom onset at 6–12 h, 31 deaths in patients with a time-of-day at symptom onset at 12–18 h and 21 deaths in patients with a time-of-day at symptom onset at 18–24 h (Kaplan–Meier estimates of mortality, 13.6%, 8.7%, 13.7% and 9.3%, respectively [log-rank test p-value = 0.30]). Cardiac deaths occurred in 68 patients: 19 deaths in patients with a time-of-day at symptom onset at 0–6 h, 12 deaths in patients with a time-of-day at symptom onset at 6–12 h, 22 deaths in patients with a time-of-day at symptom onset at 12–18 h and 15 deaths in patients with a time-of-day at symptom onset at 18–24 h (Kaplan–Meier estimates of mortality, 9.0%, 4.3%, 9.7% and 6.4%, respectively [log-rank test p-value = 0.13]). Kaplan–Meier curves of all-cause and cardiac mortality are shown in Fig. 3.

There was no association between time-of-day at symptom onset and the frequency of other clinical outcomes including nonfatal myocardial infarction, target vessel revascularization or MACE (Table 4). After adjustment in the Cox proportional hazards model (see “Methods” for variables entered into the model) there was no association between time-of-day at symptom onset and 5-year all-cause or cardiac mortality. Unadjusted and adjusted hazards ratios for mortality in 0–6 h, 12–18 h and 18–24 h time intervals versus 6–12 h time interval (reference) are shown in Table 5. Of note, an analysis including only individuals presenting within 3 h after symptom onset did also not reveal different results (Additional file 1: Table S4).

**Table 4:** Clinical outcome at 5-year follow-up

Outcome	Time-of-day				p-value
	0–6 h	6–12 h	12–18 h	18–24 h	
All-cause mortality	28 (13.6)	24 (8.7)	31 (13.7)	21 (9.3)	0.30
Cardiac mortality	19 (9.0)	12 (4.3)	22 (9.7)	15 (6.4)	0.13
Myocardial infarction	9 (3.4)	15 (5.0)	11 (5.0)	6 (2.2)	0.60
Target vessel revascularization	59 (23.4)	94 (30.4)	69 (24.6)	67 (26.8)	0.40
Major adverse cardiovascular events	81 (33.5)	117 (38.0)	95 (36.2)	83 (32.6)	0.80

Data are number of events with Kaplan-Meier estimates

**Table 5:** Association of time-of-day at symptom onset with long-term clinical outcome (results obtained from the univariable and multivariable Cox proportional hazards model)

Outcome	Risk estimate	Time-of-day				p-value
		0–6 (n = 273)	6–12 (n = 355)	12–18 (n = 309)	18–24 (n = 269)	
All-cause mortality	Unadjusted HR (95% CI)	1.525 (0.884–2.631)^1^	Ref	1.550 (0.910–2.642)^2^	1.153 (0.642–2.071)^3^	0.11^1^ 0.63^2^ 0.13^3^
	Adjusted HR (95% CI)	1.422 (0.780–2.592)^1^	Ref	1.190 (0.644–2.198)^2^	1.155 (0.587–2.275)^3^	0.25^1^ 0.58^2^ 0.68^3^
Cardiac mortality	Unadjusted HR (95% CI)	2.073 (1.006–4.271)^1^	Ref	2.190 (1.084–4.426)^2^	1.648 (0.772–3.522)^3^	0.05^1^ 0.03^2^ 0.20^3^
	Adjusted HR (95% CI)	2.083 (0.927–4.679)^1^	Ref	1.805 (0.790–4.121)^2^	1.858 (0.766–4.507)^3^	0.08^1^ 0.16^2^ 0.17^3^

The multivariable model for all-cause mortality includes hypercholesterolemia, anterolateral location of MI, time to admission, door to balloon time, presentation during office hours, age, gender, diabetes mellitus, BMI, prior MI, prior CABG, Killip class ≥ 2 at presentation, GFR, multivessel disease, no reflow after PCI, LV-EF at baseline, and infarct vessel. The multivariable model for cardiovascular mortality includes hypercholesterolemia, anterolateral location of MI, time to admission, door to balloon time, presentation during office hours, age, gender, diabetes mellitus, hypertension, prior MI, prior CABG, Killip class ≥ 2 at presentation, GFR, multivessel disease, no reflow after PCI, LV-EF at baseline, and infarct vessel

Data are hazards ratios (HR) with 95% confidence interval (CI). The 6–12 h time interval served as reference

^1^0–6 h time interval vs. reference time interval

^2^12–18 h time interval vs. reference time interval

^3^18–24 h time interval vs. reference time interval

## Discussion

The main findings of this study may be summarized as follows: (1) Time-of-day at symptom onset in patients with STEMI was not associated with initial area at risk, infarct size or amount of myocardium salvaged by PPCI. (2) Time-of-day at the symptom onset was not associated with the 5-year risk of all-cause mortality, cardiac mortality, nonfatal myocardial infarction, target vessel revascularization or MACE. (3) Time-of-day at the symptom onset was not associated with differences in the recovery of LV-EF at 6 months after PPCI.

Most organisms display intrinsic body clocks that respond to external cues and control major functions during steady state and pathologies [1, 5, 26–29]. Photic cues (light/dark pattern) are received by the retina, then signal via the retinohypothalamic tract and are processed in the hypothalamic suprachiasmatic nuclei which represents the central/master clock of the body [26]. This activates the (i) hypothalamic–pituitary–adrenal axis, which triggers release of adrenocorticotropic hormone from the cortex of the pituitary gland and (ii) the sympathetic–adrenal–medullary axis which stimulates of the adrenal medulla to produce the catecholamines. Additionally, the sympathetic nervous system (SNS) directly innervates peripheral end organs and regulates circadian rhythms locally by releasing the neurotransmitter noradrenaline. Apart from the cardiovascular system, the immune system also displays circadian variations. Blood leukocytes, for instance, are known to oscillate in terms of numbers and phenotypes during the course of a day [14, 30, 31]. In humans, monocyte and neutrophil numbers peak at around 22 h (light-to-dark transition) and show a trough at around 8 h (dark to light transition) [24, 25].

In our study, most STEMI cases occurred between 6 and 12 h. This is in line with previous findings on MI occurrence [3, 32, 33] and may be explained by the fact that plaque rupture/erosion leading to coronary occlusion is more likely during morning hours due to increased hemodynamic stress (surge in heart rate and blood pressure), thrombotic activity, i.e., platelet aggregability, and recruitment of inflammatory leukocyte from blood to plaque during this time-of-day [34–36]. Regarding leukocyte recruitment, it was recently shown in mice that the sympathetic activity increases during the active phase which raises levels of endothelial cell adhesion molecules (ICAM-1, VCAM-1, P-, and E-selectin) facilitating leukocyte recruitment to peripheral tissues (bone marrow and cremaster muscle) [36, 37]. The increased leukocyte homing and extravasation contributes to the observed lower levels of circulating blood leukocytes. Inversely, SNS activity is low during the resting phase which maintains basal levels of endothelial cell adhesion molecules to support low levels of leukocyte recruitment. This results in higher numbers of blood leukocytes during the resting phase and might offer an explanation why plaque rupture is highest during morning hours (more recruitment of inflammatory blood leukocytes to the plaque). However, in our STEMI patients blood leukocyte levels did not exhibit circadian oscillations, but were rather equally distributed throughout the day. One may speculate that MI, a strong (sympathetic) stimulus, overrides moderate circadian variations in healthy humans which are known to rely on oscillating sympathetic activity [36, 37]. Moreover, mice unlike humans are nocturnal animals and hence are active during the dark phase. Thus, findings regarding circadian rhythms in nocturnal mice may not be so simply extrapolated to diurnal rhythms in humans.

We found no association between the time-of-day at symptom onset of STEMI and scintigraphic indices such as initial area at risk, infarct size or amount of myocardium salvaged by PPCI. In this regard our findings concur with a recent study by Ammirati et al. [20]. which found no clear-cut circadian dependence of infarct size after STEMI. Other studies, however, reported larger infarct sizes for the tie-of-day at symptom onset between 6 and 12 h [6–10, 16, 17]. These studies used either peak CK (creatine kinase), peak troponin I or cardiac magnetic resonance imaging to determine the infarct size. The strength of our study rests on the serial use of SPECT imaging which represents a reliable and validated tool for the estimation of infarct size in clinical setting [38]. Moreover, paired SPECT imaging allowed us to assess other important parameters like initial myocardial area at risk and the amount of myocardium salvaged by PPCI and correlate them with the time-of-day at symptom onset. Likewise, we found no association between time-of-day at symptom onset and infarct size assessed by peak CKMB.

In the current study, we did not find a higher probability of LV function recovery for individuals with time-of-symptom onset between 6 and 12 h as compared to those between 18 and 24 h indicating that time-of-day symptom onset may not affect LV-remodeling. While human data are scarce, preclinical studies showed a circadian influence on cardiac remodeling. Similar to circadian variations in leukocyte recruitment to peripheral tissues (see above), recruitment of blood leukocytes to the infarcted heart also seems to be facilitated when injury occurs during the active phase. It was recently shown that neutrophils carry more of the chemokine receptor CXCR2 and the heart expresses more cell adhesion molecules and chemokines during the active phase of the day [14]. Consequently, exaggerated accumulation of leukocytes from the blood to the heart during the active phase may result in adverse cardiac remodeling. Similar to permanent ligation studies, ischemia/reperfusion performed at the sleep-to-wake transition also resulted in adverse cardiac remodeling in comparison to ischemia/reperfusion performed at the wake-to-sleep transition [15]. Another recent study investigated the effect of a disrupted dark–light pattern in mice on adverse cardiac remodeling after MI [39]. The authors found that diurnal rhythm disruption immediately post-MI impaired healing and exacerbated maladaptive cardiac remodeling. These effects were mainly caused by an altered innate immune response leading to an exaggerated accumulation of cardiac macrophages in the group with disrupted rhythm. However, our human data do not support these preclinical findings. Although the reasons for this discrepancy are not clear it may be speculated that (i) larger patient cohorts are needed to unmask a circadian effect, (ii) most mouse studies used permanent ligation while all our patients had spontaneously occurring STEMI and underwent revascularization, and (iii) that findings from nocturnal mice may not be directly translated to diurnal humans.

Finally, we did not find a significant association between the time-of-day at symptom onset and clinical outcome. We speculate that this may be a direct consequence of our findings which showed no association between circadian rhythms infarct size or amount of myocardium salvaged after PPCI or LV function at 6 months after STEMI. Although the statistical significance was not achieved, patients with symptom onset at 6–12 h showed the lowest rates of crude all-cause and cardiac mortality. To the best of our knowledge, this study represents the first analysis of an association between time-of-day symptom onset and 5-year outcome in patients with STEMI. All other studies have analyzed short-term in-hospital, 30-day or 1-year mortality [6–11].

Our study has several limitations. First, it was a retrospective analysis of patients presenting with STEMI treated with PPCI in tertiary care centers between 2002 and 2007 and not a dedicated trial designed to investigate the association of time-of-day at symptom onset with infarct size or clinical outcome of patients with STEMI. Time-of-day at symptom onset was documented in all individuals included in this study as standard routine care using a specific questionnaire. However, patient-reported time of symptom onset is subjective and may represent an inaccurate measure of true time of STEMI onset. In that light, it was recently reported that the biochemical onset of STEMI (Troponin T release) may occur even earlier than the patient-reported onset time [40]. Moreover, pre-infarction angina may also have occurred and contributed further to an imprecise determination of STEMI onset. Obviously, type and timing of reperfusion therapy might have affected the outcome of our study. Time-to-admission intervals differed significantly. However, adjustment for time-to-admission intervals did not impact the investigated outcomes. Third, the angiographic follow-up measurements of LV-EF were available in only 39% (n = 470) and 5-year follow-up only in 37% of the patients (n = 451). Furthermore, STEMI patients were enrolled between 2002 and 2007 and may hence with respect to stent technology and anti-platelet therapy not be the most contemporary STEMI cohort. Last, we did not have access to cardiovascular medication (or adherence) during the follow-up which might have had an impact on clinical outcome. Although undesirable, we do not believe that these limitations impact on the main findings of the study.

## Conclusions

In STEMI patients treated with PPCI, time-of-day at symptom onset was not associated with differences in final infarct size, amount of myocardium salvaged by PPCI or recovery of LV function or long-term (5-year) all-cause or cardiac mortality.

## Additional file

**Additional file 1: Table S1.** Univariate correlates of 5-year all-cause mortality. **Table S2.** Univariate correlates of 5-year cardiovascular mortality. **Table S3.** Association of time-of-day at symptom onset with area at risk, final infarct size or salvage index in individuals presenting within three hours after symptom onset. **Table S4.** Association of time-of-day at symptom onset with long-term clinical outcome (results obtained from the univariable Cox proportional hazards model) in individuals presenting within three hours after symptom-onset. **Figure S1.** Total blood leukocyte counts on admission in relation to time-of-day at symptom onset.

## Data Availability

The authors declare that the data supporting the findings of this study are available within the article and its additional information files.

## Abbreviations

BMI: body mass index
CABG: coronary artery bypass graft
CD: cardiac death
CK: creatine kinase
CKMB: creatine kinase myocardial band
GFR: glomerular filtration rate
LAD: left anterior descending artery
LCX: left circumflex artery
LM: left mainstem
LV: left ventricular
LV-EF: left ventricular ejection fraction
MACE: major adverse cardiovascular events
MI: myocardial infarction
PPCI: primary percutaneous coronary intervention
RCA: right coronary artery
SPECT: single photon emission computed tomography
SNS: sympathetic nervous system
STEMI: ST-segment elevation myocardial infarction
