# Supplementary material for: Time-of-day at symptom onset was not associated with infarct size and long-term prognosis in patients with ST-segment elevation myocardial infarction
Source: J Transl Med. 2019 May 29;17:180. doi: 10.1186/s12967-019-1934-z (PMC6542088; doi:10.1186/s12967-019-1934-z)
Supplement: Supplementary file 1 — Additional file 1: Table S1. Univariate correlates of 5-year all-cause mortality. Table S2. Univariate correlates of 5-year cardiovascular mortality. Table S3. Association of time-of-day at symptom onset with area at risk, final infarct size or salvage index in individuals presenting within three hours after symptom onset. Table S4. Association of time-of-day at symptom onset with long-term clinical outcome (results obtained from the univariable Cox proportional hazards model) in individuals presenting within three hours after symptom-onset. Figure S1. Total blood leukocyte counts on admission in relation to time-of-day at symptom onset. [file 12967_2019_1934_MOESM1_ESM.docx]

**Time-of-day at symptom onset was not associated with infarct size and long-term prognosis in patients with ST-segment elevation myocardial infarction**

Additional file 1

**Additional file 1: Table S1**. Univariate correlates of 5-year all-cause mortality.

| Characteristic | | Hazard ratio | 95% CI | p-value |
| --- | --- | --- | --- | --- |
| Age | | 1.074 | 1.055-1.092 | <0.001 |
| Female gender | | 2.000 | 1.342-2.982 | 0.001 |
| Diabetes mellitus | | 2.242 | 1.493-3.368 | <0.001 |
| BMI | | 0.931 | 0.881-0.983 | 0.01 |
| Hypertension | | 1.544 | 0.972-2.453 | 0.07 |
| Hypercholesterolemia | | 0.974 | 0.661-1.435 | 0.89 |
| Current smoker | | 0.685 | 0.457-1.026 | 0.07 |
| Prior MI | | 2.338 | 1.491-3.666 | <0.001 |
| Prior CABG | | 2.752 | 1.389-5.451 | 0.004 |
| Anterolateral location of MI | | 1.508 | 0.994-2.288 | 0.05 |
| Killip class ≥2 | | 3.199 | 2.178-4.699 | <0.001 |
| ST-segment resolution | | 1.034 | 0.595-1.799 | 0.91 |
| GFR | | 0.973 | 0.966-0.981 | <0.001 |
| Time to admission | | 1.018 | 0.988-1.049 | 0.25 |
| Door to balloon-time | | 0.848 | 0.653-1.100 | 0.21 |
| Multivessel disease | | 2.541 | 1.545-4.179 | <0.001 |
| Baseline TIMI flow grade | |  | | |
|  | 0 | Ref | | |
|  | 1 | 0.696 | 0.344-1.409 | 0.31 |
|  | 2 | 0.679 | 0.393-1.170 | 0.16 |
|  | 3 | 1.175 | 0.727-1.901 | 0.51 |
| No reflow | | 1.781 | 1.113-2.852 | 0.02 |
| Type of intervention | |  | | |
|  | Balloon angioplasty | Ref | | |
|  | Stenting | 0.721 | 0.446-1.166 | 0.18 |
| LV-EF at baseline | | 0.962 | 0.946-0.979 | <0.001 |
| Infarct vessel | |  |  |  |
|  | LM | Ref | | |
|  | LAD |  |  |  |
|  | LCx | 0.626 | 0.342-1.146 | 0.13 |
|  | RCA | 0.679 | 0.435-1.059 | 0.09 |
|  | CABG | 2.655 | 1.143-6.167 | 0.03 |
| Time of symptom onset | |  | | |
|  | 6-12 h | Ref | | |
|  | 12-18 h | 1.550 | 0.910-2.642 | 0.11 |
|  | 18-24 h | 1.153 | 0.642-2.071 | 0.63 |
|  | 0-6 h | 1.525 | 0.884-2.631 | 0.129 |
| Presentation during office hours | | 0.871 | 0.592-1.280 | 0.48 |

Data are presented as hazard ratios (95% confidence interval) with time-of-day at symptom onset 0-6h defined as reference. *BMI*, body mass index; *CABG*, coronary artery bypass graft; *CI*, confidence interval; *CV*, cardiovascular*; GFR*, glomerular filtration rate; *HR*, hazard ratio*; LV-EF*, left ventricular ejection fraction; *LAD*, left anterior descending artery; *LCX*, left circumflex artery; *LM*, left mainstem; *MI*, myocardial infarction; *RCA*, right coronary artery.

**Additional file 1: Table S2**. Univariate correlates of 5-year cardiovascular mortality.

| Characteristic | | Hazard ratio | 95% CI | p-value |
| --- | --- | --- | --- | --- |
| Age | | 1.081 | 1.057-1.104 | <0.001 |
| Female gender | | 2.417 | 1.491-3.918 | <0.001 |
| Diabetes mellitus | | 2.404 | 1.462-3.952 | 0.001 |
| BMI | | 0.943 | 0.882-1.009 | 0.09 |
| Hypertension | | 2.040 | 1.094-3.806 | 0.03 |
| Hypercholesterolemia | | 0.994 | 0.616-1.606 | 0.98 |
| Current smoker | | 0.619 | 0.372-1.028 | 0.06 |
| Prior MI | | 2.666 | 1.556-4.570 | <0.001 |
| Prior CABG | | 3.858 | 1.845-8.069 | <0.001 |
| Anterolateral location of MI | | 1.416 | 0.852-2.354 | 0.18 |
| Killip class ≥2 | | 3.596 | 2.233-5.790 | <0.001 |
| ST-segment resolution | | 0.873 | 0.466-1.632 | 0.67 |
| GFR | | 0.976 | 0.967-0.985 | <0.001 |
| Time to admission | | 1.030 | 0.994-1.068 | 0.10 |
| Door to balloon-time | | 0.851 | 0.617-1.175 | 0.33 |
| Multivessel disease | | 2.936 | 1.540-5.598 | 0.001 |
| Baseline TIMI flow grade | |  | | |
|  | 0 | Ref | | |
|  | 1 | 0.613 | 0.240-1.567 | 0.31 |
|  | 2 | 0.881 | 0.473-1.643 | 0.69 |
|  | 3 | 1.168 | 0.636-2.144 | 0.62 |
| No reflow | | 1.872 | 1.055-3.322 | 0.03 |
| Type of intervention | |  | | |
|  | Balloon angioplasty | Ref | | |
|  | Stenting | 0.625 | 0.352-1.111 | 0.11 |
| LV-EF at baseline | | 0.957 | 0.937-0.977 | <0.001 |
| Infarct vessel | |  |  |  |
|  | LM | Ref | | |
|  | LAD |  |  |  |
|  | LCx | 0.917 | 0.462-1.818 | 0.80 |
|  | RCA | 0.732 | 0.415-1.292 | 0.28 |
|  | CABG | 4.540 | 1.898-10.859 | 0.001 |
| Time of symptom onset | |  | | |
|  | 6-12 h | Ref | | |
|  | 12-18 h | 2.190 | 1.084-4.426 | 0.029 |
|  | 18-24 h | 1.648 | 0.772-3.522 | 0.197 |
|  | 0-6 h | 2.073 | 1.006-4.271 | 0.048 |
| Presentation during office hours | | 0.779 | 0.484-1.254 | 0.30 |

Data are presented as hazard ratios (95% confidence interval) with time-of-day at symptom onset 0-6h defined as reference. *BMI*, body mass index; *CABG*, coronary artery bypass graft; *CI*, confidence interval; *CV*, cardiovascular*; GFR*, glomerular filtration rate; *HR*, hazard ratio*; LV-EF*, left ventricular ejection fraction; *LAD*, left anterior descending artery; *LCX*, left circumflex artery; *LM*, left mainstem; *MI*, myocardial infarction; *RCA*, right coronary artery.

**Additional file 1: Table S3**. Association of time-of-day at symptom onset with area at risk, final infarct size or salvage index in individuals presenting within three hours after symptom onset.

| Risk estimate | Time-of-day at symptom onset (hours) | | | | p-value |
| --- | --- | --- | --- | --- | --- |
|  | 0-6h  (n=66) | 6-12h  (n=160) | 12-18h  (n=139) | 18-24h  (n=91) |  |
| Area at risk > median | | | | | |
| OR (95% CI) | 0.864  (0.486-1.534)^1^ | Ref | 1.177  (0.746-1.856)^2^ | 1.189  (0.710-1.993)^3^ | ^1^ 0.62  ^2^ 0.48  ^3^ 0.51 |
| Final infarct size > median | | | | | |
| OR (95% CI) | 0.801  (0.451-1.423)^1^ | Ref | 0.729  (0.462-1.150)^2^ | 1.010  (0.603-1.691)^3^ | ^1^ 0.45  ^2^ 0.17  ^3^ 0.97 |
| Salvage index > median | | | | | |
| OR (95% CI) | 1.148  (0.634-2.079)^1^ | Ref | 1.156  (0.726-1.840)^2^ | 1.118  (0.655-1.908)^3^ | ^1^ 0.65  ^2^ 0.54  ^3^ 0.68 |

Data are presented as odds ratios (OR) with 95% confidence interval (CI) with time-of-day at symptom onset 0-6h serving as reference (Ref). ^1^ indicates 0-6h time interval vs. reference time interval, ^2^ indicates 12-18h time interval vs. reference time interval, ^3^ indicates 18-24h time interval vs. reference time interval.

**Additional file 1: Table S4**. Association of time-of-day at symptom onset with long-term clinical outcome (results obtained from the univariable Cox proportional hazards model) in individuals presenting within three hours after symptom-onset.

| Outcome | Risk estimate | Time-of-day | | | | p-value |
| --- | --- | --- | --- | --- | --- | --- |
|  |  | 0-6  (n=66) | 6-12  (n=160) | 12-18  (n=139) | 18-24  (n=91) |  |
| All-cause mortality | HR (95%CI) | 1.597  (0.568-4.489)^1^ | Ref | 1.712  (0.732-4.006)^2^ | 0.567  (0.154-2.096)^3^ | ^1^ 0.37  ^2^ 0.22  ^3^ 0.40 |
| Cardiac mortality | HR (95%CI) | 2.423  (0.606-9.692)^1^ | Ref | 2.387  (0.718-7.929)^2^ | 1.285  (0.288-5.743)^3^ | ^1^ 0.21  ^2^ 0.16  ^3^ 0.74 |

Data are hazards ratios (HR) with 95% confidence interval (CI). The 6-12h time interval served as reference (Ref). ^1^ indicates 0-6h time interval vs. reference time interval, ^2^ indicates 12-18h time interval vs. reference time interval, ^3^ indicates 18-24h time interval vs. reference time interval.


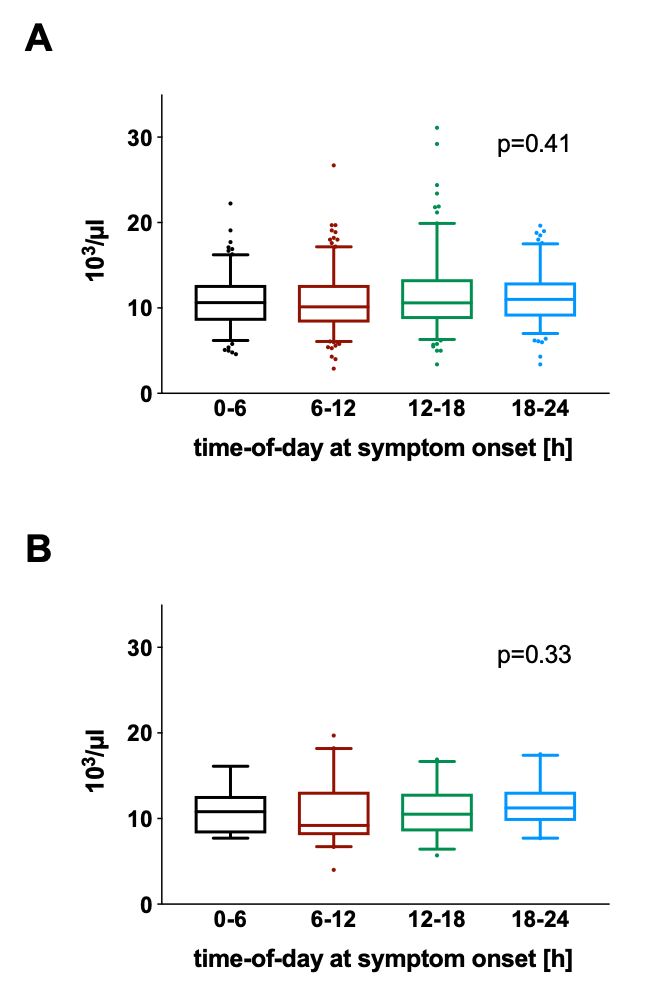


**Additional file 1: Figure S1**. Total blood leukocyte counts on admission in relation to time-of-day at symptom onset. (**A**) all available patients (n=660 (55%)); (**B**) only patients (n=106 (9%)) with total ischemic time (time to admission + time to intervention) ≤3h. Data are median and interquartile range. Kruskal-Wallis-test.
